# Detection of Occult Metastases in Patients with T1 and T2 Stage Lower Lip Squamous Cell Carcinomas after Positive Lymphoscintigraphy

**DOI:** 10.3390/diagnostics10020097

**Published:** 2020-02-11

**Authors:** Mergime Prekazi Loxha, David Stubljar, Tomislav Jukic, Sinan Rusinovci

**Affiliations:** 1 Department of Maxillofacial Surgery, Faculty of Medicine Pristina, 10000 Pristina, Kosovo; 2 Department of Research and Development, In-Medico, 8330 Metlika, Slovenia; 3 Department of Internal medicine, History of Medicine and Medical Ethics, Faculty of Medicine, Josip Juraj Strossmayer University of Osijek, 31000 Osijek, Croatia

**Keywords:** squamous cell carcinoma, lower lip, lymphoscintigraphy, metastasis, sentinel lymph node biopsy

## Abstract

The aim of this study was to detect lower lip squamous cell carcinomas (SCC) that had metastasized to the lymph nodes and to evaluate if neck dissection was necessary for patients with T1 or T2-stage lip cancer after a sentinel lymph node biopsy (SLNB). The study was conducted as a prospective clinical study to detect occult neck metastases in patients with T1 or T2 stage SCC of the lower lip. Thirty-one patients were eligible and underwent echo-ultrasound, computer tomography, magnetic resonance and lymphoscintigraphy (LSG) as diagnostic procedures. LSG was performed on the same day as the surgical procedure, after intradermal injection of 37 Mbq Tc99m-Sn-colloid/mL at four peritumoral sites. In patients with positive LSG results, the sentinel lymph nodes were extracted surgically. The risk factors for cancer development were sun exposure and smoking. The highest accuracy for detecting lymph node enlargements was achieved with magnetic resonance imaging (MRI; 80.7%). LSG showed excellent sensitivity (100%) and negative predictive value (NPV; 100%). Overall, occult metastases were diagnosed with an SLNB in eight (25.8%) patients. According to the results, with great caution, we suggest that an SLNB is reasonable to initiate only for patients with positive sentinel nodes by positive LSG, to be used as a lower morbidity approach for selected patients with T1 and T2 stage cancers.

## 1. Introduction

Squamous cell carcinoma (SCC) of the lower lip accounts for 25% of all oral cancers [1,2]. It is an epithelial malignant, infiltrating and destructive tumor with metastatic potential that can invade the deep muscle and mandible and can metastasize to the neck lymph nodes or blood [3]. Regional metastases rates vary and could be present in 5% to 20% of cases, and their presence reduces the prognosis to 50% [4]. SCC of the lips can develop at any position along the upper or lower lip, but 90% of all cases involve the lower lip [5]. It is more frequent in male than female patients; patients aged over 45 years; those with chronic sun exposure, and chronic smoking and alcohol-drinking habits [1,6]; and in patients with accompanied systemic lupus erythematosus [7,8].

SCC tumors are not acknowledged as fatal [9,10,11,12]. Surgical excision of a primary tumor achieves a 5-year cure rate of 92%, with an overall recurrence rate of only 8% [13]. The mean survival rate of patients is 90% at 2 years and 83% at 5 years. However, patients with a T3 or T4 stage SCC and those with metastases have an unfavorable prognosis [3]. The five-year overall survival of patients with regional metastases ranges from 25% to 70% [14]. After treatment, patients must be examined and followed-up for at least 5 years to find possible recurrences and potential regional metastases. Almost 75% of metastasis appear in the first year after surgery, a period in which examinations must be periodic, more frequent, and accurate [3].

Surgical procedures play an important treatment role in SCC management. Surgery includes full tumor excision, lymph node dissection, and simultaneous reconstruction [15,16]. One of the characteristics of lips SCC is the extension of the tumor, which can manifest either towards the surface, in depth or in both directions. For instance, the occult metastases rates of a T2 stage lip cancer were reported in 15% to 35% of cases [17]. In such cases, elective neck dissection with excision of the primary tumor is the treatment of choice [18]. However, not every patient should be recommended for elective neck dissection. The presence of lymph node metastases is the main prognostic factor; however, most diagnostic methods used to detect susceptible metastases in the neck are not accurate. Computer tomography (CT), magnetic resonance imaging (MRI) and ultrasound can detect lymph nodes but not metastases. Therefore, sentinel lymph node biopsy (SLNB) is the most advisable option to diagnose metastases in the neck lymph nodes, especially in patients with N0 [17], and it may help in the treatment of these patients [19]. SLNB, as one of the procedures, is standard care for melanoma [20]. However, the data on patients with nonmelanoma skin cancers (NMSC) are sparse [21,22]. The patients with NMSC, such as SCC, show lymph node metastasis [9,23,24], but to our knowledge there has been no report of a relatively large number of patients followed in a prospective study. Therefore, it is vital to collect data on SLNB in SCC cases and examine its accuracy.

In the current study, we focused on analyzing patients who were suffering from stage T1 and T2 of SCC of the lower lip. Moreover, metastatic tumors through a 2-year follow-up period were also detected using SLNB. Thus, our study aimed to analyze lower lip SCC tumors that had metastasized to the lymph nodes, to evaluate the accuracy of diagnostic tools for lymph node enlargements (echo-ultrasound, magnetic resonance imaging (MRI), CT and lymphoscintigraphy (LSG)), and show if elective or supraomohyoid neck dissection was necessary for patients with T1 or T2-stage lip cancer, to evaluate the applicability of the SLNB concept for T1 and T2 SCC of the lower lip.

## 2. Materials and Methods

The study was conducted as a prospective clinical study to detect occult neck metastases in patients with T1 or T2 stage squamous cell carcinoma (SCC) of the lower lip. Patients were treated at the Department of Maxillofacial Surgery at the University Clinical Centre of Kosovo and followed for 2 years. The research was conducted in full accordance with the medical protocols of the Declaration of Helsinki. Patients gave their written consent for collaboration in the study. The Institutional Ethics Committee of the Faculty of Medicine, University of Pristina approved the study design (document Nr.1551, 30.03.2010).

The inclusion criteria for patients to participate were aged 18 years and older, detected SCC of the lower lip with TNM classification cT1 and cT2 and with an indication for surgical intervention. Patients were excluded when treated with radiotherapy before surgery, with recurrent carcinoma, or with stage T3 and T4 lower lip carcinoma. Overall, 31 patients were eligible for inclusion in the analysis. 

Eligible patients underwent echo-ultrasound, computer tomography (CT), magnetic resonance (MRI) and lymphoscintigraphy (LSG) as diagnostic procedures. At enrolment, clinically relevant data (including exposure to potential risk factors such as sun exposure, alcohol and tobacco use, and family history) were collected. Clinical examination of the oral cavity and oropharynx was performed in all patients. LSG of the neck was performed to detect lymph nodes in the neck. LSG was performed on the same day as surgical procedure, after intradermal injection of 37 Mbq Tc99m-Sn-colloid/mL at four peritumoral sites. In patients with positive LSG results, sentinel lymph nodes were extracted surgically. The extracted nodes were sent for histopathological analysis to confirm cancer metastases.

## 3. Results

**Figure 1:**
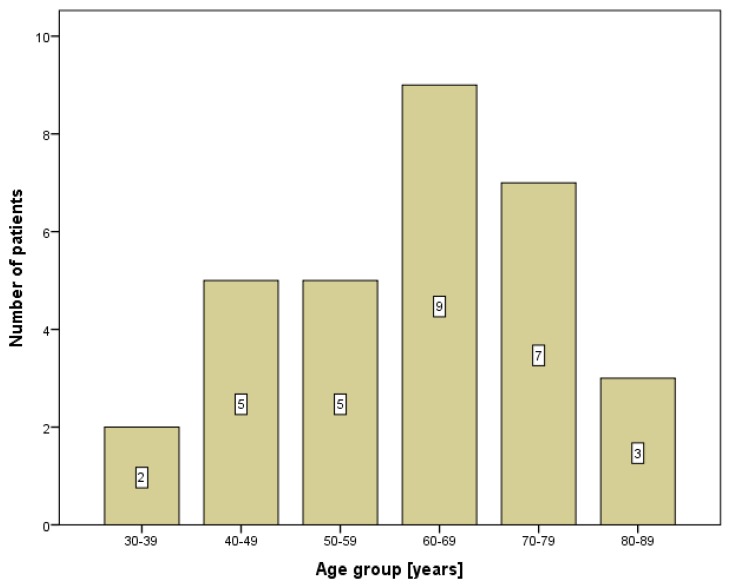
The age distribution of patients with lower lip cancer.

Out of the 31 patients enrolled in the study, the majority were men (*n* = 28; 90.3%). The mean age of the patients in the present study was 61.6 ± 13.4 years, as shown in Table 1. More than half of the patients (*n* = 19; 61.3%) were 60 years of age and older. According to the age distribution, a positive diagnosis of SCC of the lower lip was detected at two peaks. Most often, the cancer occurred in patients aged 60–69 and 70–79 years, as shown in Figure 1. Almost half (45.2%) of all patients were farmers by profession.

The characteristics of the cancers are presented in Table 2. The majority of patients (*n* = 22; 71.0%) had stage T1 SCC. Lymph node enlargements were discovered in 10 patients. Approximately half of the patients suffered the disease for more than 1 year. The most frequent risk factors for cancer development were sun exposure and smoking; meanwhile, alcohol use and family history were not so frequent. There were no statistical differences in patients’ basic characteristics and cancer characteristics between genders (data not shown).

Positive lymph enlargements were detected in 10 patients (32.3%). Meanwhile, positive ultrasound, MRI, CT and LSG were detected in 13, 12, 2 and 21 patients, respectively, as shown in Table 2. Among 21 positive LSG patients, the majority had lymph node enlargements in both submental and submandibular regions. No potential risk factors were detected that could be statistically associated with the prediction of lymph node enlargements, as shown in Table 3.

**Figure 2:**
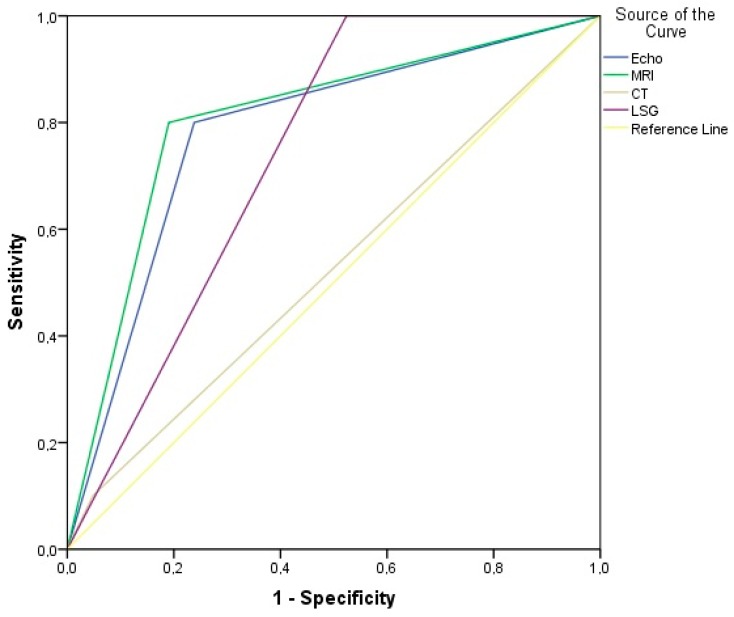
The ROC curve analysis to predict lymph node enlargement with four different diagnostic tools (echo-ultrasound, MRI, CT and LSG).

We were also interested in observing which diagnostic tool could potentially predict positive lymph node enlargement. Table 4 and Figure 2 present the values of receiver operating characteristic curve (ROC) analysis for detecting lymph node enlargement. The highest accuracy was found with MRI (80.7%). Meanwhile, LSG showed excellent sensitivity (100%) and negative predictive value (NPV; 100%) value but due to a high count of false-positive results (*n* = 11 cases) was lower on specificity and PPV.

Afterwards, a sentinel node biopsy (SNB) was performed on 21 patients with a positive LSG finding due to the excellent sensitivity to detect true positive patients. Occult metastases using an SLNB were diagnosed in eight patients (38.1%) with positive LSG results. All eight cases were confirmed by histopathology and had a pathology duration of over 1 year (*p* = 0.003), leading to an overall occult metastases rate of 25.8% out of all 31 enrolled patients. Higher age and lymph node enlargements were the only two risk factors that showed statistically significant association with occult metastases, as shown in Table 5.

## 4. Discussion

Patients who were suffering from stage T1 and T2 SCC of the lower lip were followed-up over 2 years. The current study aimed to detect lower lip SCC tumors that had metastasized to the lymph nodes, to evaluate the accuracy of the diagnostic tools for lymph node enlargements (echo-ultrasound, MRI, CT and LSG) and to answer the thesis of whether elective or supraomohyoid neck dissection was necessary for patients with T1 or T2-stage lip cancer after evaluation of the SLNB.

The study included 31 patients whose demographic characteristics with a mean age of 61.6 years were comparable with the literature [25,26]. The majority were men and had been exposed to the sun and had smoking habits, which coincide with the knowledge of potential risk factors [1,6]. There are many risk factors for developing lip cancer, including higher age (especially for 60–70-year-olds), male gender, chronic exposure to sun radiation, tobacco and alcohol consumption, viral factors such as Human Papilloma Virus (HPV) 16 and 24, and Herpes Virus (HSV) 1 and 2 and accompanied autoimmune diseases and immunosuppressant drugs [6,27,28]. Farmers were also detected as a risk group of people for cutaneous SCC. This might be explained due to their sun exposure while working outside in the fields. In a case report by Nguyen et al. [29], a patient with SCC was also a farmer and often worked in sunlight. He had also smoked and consumed a great deal of alcohol for a long time.

Our results showed that the majority of patients (*n* = 22; 71.0%) in the current report had T1 stage SCC. Lymph node enlargements (Nc1) were discovered in 10 patients (32.3%). Meanwhile, positive ultrasound, MRI, CT and LSG were detected in 13, 12, 2 and 21 patients, respectively. Therefore, ROC analysis showed the highest accuracy for detecting Nc1 with MRI (80.7%), but LSG showed excellent sensitivity (100%) and negative predictive value (100%) for Nc1. That means that LSG was the test with the highest probability for detecting patients with true positive lymph node enlargements (Nc1). With different values for the diagnostic tools, we can confirm the findings from the literature that several factors may influence a misdiagnosis: a low incidence of lymph node metastasis, slow diffusion, deep metastasis, lymph node jump, fixation of the lymph node metastasis to the mandibular periosteum, and previous radiotherapy. Several factors are directly correlated to lymph node involvement, including tumor size and differentiation [12,13,30,31,32].

An SLNB was thus performed in all 21 patients with a positive LSG finding. Occult metastases were diagnosed in eight (38.1%) patients leading to an overall occult metastasis in 25.8% out of 31 enrolled patients. Interestingly, two (25%) out of eight patients had Nc0 and had developed metastases. Moreover, out of eight patients, six (75%) had a T1 size tumor and two (25%) did not have lymph node enlargements. The question arises: should patients with T1 and Nc0 avoid neck dissection? The management of patients with SCC with a clinically negative neck remains debatable, and the majority of clinicians prefer an elective neck dissection instead of a “wait-and-see” strategy due to the high rates of occult metastases [33]. However, almost 70% (*n* = 21) of our patients had N0 and could be theoretically over-treated with a selective neck dissection [34].

Diagnostic developments have led to more extensive use of SLNB [35]. In modern surgical treatment, the presence of regional lymph node metastases is evaluated by the identification and examination of the sentinel lymph nodes [33,36]. The concept of an SLNB provides the possibility of accurate pathological node staging, whilst minimizing the invasiveness of the procedure and its associated morbidity. In addition, preoperative LSG and intraoperative detection have the additional advantage of identifying aberrant drainage pathways [34,37]. In the present study, 21 patients had positive findings but only eight were truly diagnosed. Therefore, SLNB shows the benefits of concentrating only on the relevant nodes for pathological examination. This selection allows for a more in-depth evaluation of patients and the small number of sentinel nodes, who truly develop occult metastases [37].

The sensitivity of the SLNB for head and neck cancer varies in the literature between 75% and 100%. This has to be compared with the rate of regional recurrence after a selective neck dissection, which is recorded in the literature as between 6% and 30% [38,39,40]. In the present study, we did not calculate the sensitivity of SLNB but in a study by Sagheb et al. [41] the sensitivity was shown to be 75%. Although further studies are necessary to confirm the results, patients with cN0 and early-stage SCC may benefit from an SLNB by avoiding the morbidity of a neck dissection. Although a selective neck dissection is less invasive than a radical dissection, high morbidity for the patients does exist, including shoulder dysfunction, contour changes, pain and lower lip paresis [42,43,44]. Although selective neck dissection has proven reliability and worldwide acceptance, it is an extended surgery compared to SLNB, involving a longer surgical time, higher costs and greater morbidity. Functional outcomes and postoperative complications following an SLNB are also significantly better than after a selective neck dissection [45,46]. In a study by Civantos [34], the authors evaluated the NPV for SLNB. For T1 lesions, the NPV was 100%. The authors concluded that SLNB as a procedure was more suited for smaller lesions, and with an overall NPV of 96% for T1 or T2 N0 oral SCC, they correctly predicted a pathologically negative neck in 96% of patients. Thus, a negative SLNB would only result in the neck of 4% of patients.

Modern diagnostic developments and reports could enhance the clinical application of SLNB. We could argue that the presented results suggest that if SLNB is applied initially in an appropriately low-risk group, the procedure provides reasonable results. The effect of the procedure on the smaller group of only eight true positives is also an issue because the pathologic status of the sentinel node is sometimes not known until days after surgery. Given that the “watch-and-see” approach persists for selected T1 and T2 lesions and that some patients have circumstances that make the moderate morbidities of neck dissection unacceptable, it is likely that there may be a role for SLNB as a replaceable option. In conclusion, we suggest, but with speculation and great caution, that the SLNB is reasonable to initiate only for patients with positive sentinel nodes by positive LSG, as a lower morbidity approach for selected patients with T1 and T2 stage cancers.

However, our study had some limitations. A limitation was that the SLNB might have changed the way the neck dissection was performed. Our study design may actually lead to an underestimation of the accuracy of this technique relative to selective neck dissection, since the metastatic tumor can be left behind after an SLNB, thus requiring a further neck dissection. Such an issue was not addressed in our study design. Therefore, we should have followed our patients for a much longer time than just 2 years. Other limitations include the fact that we only detected eight cases of metastatic lymph nodes. The study presents only a small sample size of cases, so in the future a much larger study period with a much more extended follow-up period will be necessary. We have also not evaluated the predictive values for SLNB as a treatment option compared to neck dissection. In the future, more research should be conducted on this aspect.

## 5. Conclusions

Minimal damage of tissue and motoric functions are approaches that are becoming commonplace to reduce surgical morbidity. Neck dissection has always been a procedure that has resulted in high morbidities for the patients. SLNB is thus a less invasive approach and provides timely information regarding the status of the neck, and it is likely to be an attractive option for patients and physicians. Although further studies are necessary to confirm the results, patients with cN0 and early T1 or T2 stage SCC may benefit from an SLNB by avoiding the morbidity of a neck dissection. However, according to our results, with great caution, we suggest that SLNB is reasonable to initiate only for patients with positive sentinel nodes by positive LSG, as a lower morbidity approach for selected patients with T1 and T2 stage cancers.

## Figures and Tables

**Table 1:** The basic characteristics of patients with lower lip cancer.

	Patients (*n* = 31)
Age (years)	61.6 ± 13.4
Gender	
M/F	28 (90.3%)/3 (9.7%)
Profession	
Farmer	14 (45.2%)
Housewife	1 (3.2%)
Machinist	3 (9.7%)
Pensioner	6 (19.4%)
Physical worker	6 (19.4%)
Police officer	1 (3.2%)

M—male, F—female.

**Table 2:** The characteristics of squamous cell carcinoma (SCC) of the lower lip and diagnostic procedure findings.

	Patients (*n* = 31)
TNM classification	
Tc1Nc0M0	16 (51.6%)
Tc1Nc1M0	6 (19.4%)
Tc2Nc0M0	5 (16.1%)
Tc2Nc1M0	4 (12.9%)
Tumor size	
T1	22 (71.0%)
T2	9 (29.0%)
Lymph node enlargement	10 (32.3%)
Risk factors	
Sun exposure	15 (48.4%)
Smoking	22 (71.0%)
Alcohol	6 (19.4%)
Family history	5 (16%)
Duration of pathology	
Under 1 year	14 (45.2%)
Over 1 year	17 (54.8%)
Positive echo	13 (41.9%)
Positive MRI	12 (38.7%)
Positive CT	2 (6.5%)
Positive LSG	21 (67.7%)
Submental region	6 (28.6%)
Submandibular region	2 (9.5%)
Both regions	13 (61.9%)

Echo—echo-ultrasound, MRI—magnetic resonance, CT—computer tomography; LSG—lymphoscintigraphy.

**Table 3:** The potential risk factors for lymph node enlargements.

	No Enlargement (*n* = 21)	Lymph Node Enlargement (*n* = 10)	*p*-Value
Age (years)	60.2 ± 12.7	64.6 ± 15.1	0.407
Gender			
M/F	20/1	8/2	0.180
Profession			0.960
Farmer	9 (42.9%)	5 (50.0%)	
Housewife	1 (4.8%)	0	
Machinist	2 (9.5%)	1 (10.0%)	
Pensioner	4 (19.0%)	2 (20.0%)	
Physical worker	4 (19.0%)	2 (20.0%)	
Police officer	1 (4.8%)	0	
Tumor size			0.353
T1	16 (76.2%)	6 (60.0%)	
T2	5 (23.8%)	4 (40.0%)	
Sun exposure	10 (47.6%)	5 (50.0%)	0.901
Smoking	16 (76.2%)	6 (60.0%)	0.353
Alcohol	5 (23.8%)	1 (10.0%)	0.363
Family history	4 (19.0%)	1 (10.0%)	0.522
Duration of pathology			0.052
Under 1 year	12 (57.1%)	2 (20.0%)	
Over 1 year	9 (42.9%)	8 (80.0%)	

**Table 4:** The values of the receiver operating characteristic curve (ROC) analysis to detect lymph node enlargements (calculated area under curve, sensitivity, specificity and predictive values) for different diagnostic tools.

	Lymph Node Enlargement (*n* =10)	AUC (95% CI)	Sn	Sp	PPV	NPV	Accuracy	*p*-Value
Echo-ultrasound	8 (80%)	0.781 (0.600–0.962)	80.0%	86.2%	61.5%	88.9%	77.4%	0.013
MRI	8 (80%)	0.805 (0.629–0.980)	80.0%	81.0%	66.7%	89.5%	80.7%	0.007
CT	1 (10%)	0.526 (0.302–0.750)	10.0%	95.2%	50.0%	69.0%	67.7%	0.816
LSG	10 (100%)	0.738 (0.567–0.909)	100%	47.6%	47.6%	100%	64.5%	0.035

MRI—magnetic resonance, CT—computer tomography; LSG—lymphoscintigraphy, AUC—area under curve, CI—confidence interval, Sn—sensitivity, Sp—specificity, PPV—positive predictive value, NPV—negative predictive value.

**Table 5:** The predictive values for risk factor with calculated odds ratios (OR) with a 95% confidential interval (CI) for neck metastases.

	No Metastases (*n* = 23)	Metastases (*n* = 8)	B	OR *	95% CI		*p*-Value
Age	61.6 ± 13.4	63.6 ± 14.7	0.138	1.148	1.030	1.280	0.013
Gender M/F	22/1 (95.7%/4.3%)	6/2 (75.0%/25.0%)	−1.992	0.136	0.010	1.772	0.128
Profession			−0.109	0.896	0.559	1.436	0.649
Farmer	10 (43.5%)	4 (50.0%)					
Housewife	1 (4.3%)	0					
Machinist	2 (8.7%)	1 (12.5%)					
Pensioner	4 (17.4%)	2 (25.0%)					
Physical worker	5 (21.7%)	1 (12.5%)					
Police officer	1 (4.3%)	0					
TNM			0.377	1.458	0.711	2.990	0.303
Tc1Nc0M0	14 (60.9%)	2 (25.0%)					
Tc1Nc1M0	2 (8.7%)	4 (50.0%)					
Tc2Nc0M0	5 (21.7%)	0					
Tc2Nc1M0	2 (8.7%)	2 (25.0%)					
Tumor size			−0.272	0.762	0.122	4.751	0.771
T1	16 (69.6%)	6 (75.0%)					
T2	7 (30.4%)	2 (25.0%)					
Lymph node enlargement	2 (8.7%)	6 (75.0%)	2.657	14.250	2.069	98.140	0.007
Risk factors							
Sun exposure	10 (43.5%)	5 (62.5%)	0.773	2.167	0.415	11.302	0.359
Smoking	18 (78.3%)	4 (50.0%)	−1.281	0.278	0.051	1.526	0.141
Alcohol	5 (21.7%)	1 (12.5%)	−0.665	0.514	0.051	5.221	0.574
Family history	3 (13.0%)	2 (25.0%)	0.799	2.222	0.298	16.558	0.436

M—male, F—female. * odds ratios (OR) were calculated with logistic regression, where the presence of metastasis presented the dependent variable. B value was determined as a correlation coefficient between the independent and dependent variables.
